# Cross-sectional imaging of acute gynaecologic disorders: CT and MRI findings with differential diagnosis—part II: uterine emergencies and pelvic inflammatory disease

**DOI:** 10.1186/s13244-019-0807-6

**Published:** 2019-12-20

**Authors:** Pietro Valerio Foti, Massimo Tonolini, Valeria Costanzo, Luca Mammino, Stefano Palmucci, Antonio Cianci, Giovanni Carlo Ettorre, Antonio Basile

**Affiliations:** 1 Department of Medical Surgical Sciences and Advanced Technologies, Radiology I Unit, University Hospital “Policlinico-Vittorio Emanuele”, Via Santa Sofia 78, 95123 Catania, Italy; 2 Department of Radiology, “Luigi Sacco” University Hospital, Via G.B. Grassi 74, 20157 Milan, Italy; 3 Department of General Surgery and Medical-Surgical Specialties, Institute of Obstetrics and Gynecology, University of Catania, Catania, Italy

**Keywords:** Gynaecologic emergencies, Uterus, Pelvic inflammatory disease, Computed tomography (CT), Magnetic resonance imaging (MRI)

## Abstract

Due to the growing use of cross-sectional imaging in emergency departments, acute gynaecologic disorders are increasingly diagnosed on urgent multidetector computed tomography (CT) studies, often requested under alternative presumptive diagnoses in reproductive-age women. If clinical conditions and state-of-the-art scanner availability permit, magnetic resonance imaging (MRI) is superior to CT due to its more in-depth characterisationof abnormal or inconclusive gynaecological findings, owing to excellent soft-tissue contrast, intrinsic multiplanar capabilities and lack of ionising radiation.

This pictorial review aims to provide radiologists with a thorough familiarity with gynaecologic emergencies by illustrating their CT and MRI appearances, in order to provide a timely and correct imaging diagnosis. Specifically, this second instalment reviews with examples and emphasis on differential diagnosis the main non-pregnancy-related uterine emergencies (including endometrial polyps, degenerated leiomyomas and uterine inversion) and the spectrum of pelvic inflammatory disease.

## Key points

- The main uterine emergencies include bleeding endometrial lesions, degenerated leiomyomas and uterine inversion
- Identification of central fibrous core and intratumoural cysts suggest endometrial polyp over carcinoma
- The rare uterine inversion appears at MRI as U-shaped uterus with a depressed fundus and “bulls-eye” transverse configuration.
- The PID spectrum encompasses cervicitis, endometritis, salpingitis, pyosalpinx and tubo-ovarian abscess (TOA)
- Identification of fallopian tube dilatation allows differentiation of TOA from complex adnexal tumour

## Introduction

Although transabdominal and transvaginal ultrasound (US) is the ideal technique for initial investigation of women with suspected genital disorders, in the emergency department (ED), the use of cross-sectional imaging is steadily growing. As a result, nowadays radiologists increasingly encounter unsuspected female genital diseases on urgent CT studies requested under alternative presumptive diagnoses. Therefore, familiarity with these conditions and their imaging appearances is required to avoid missing or misinterpreting clinically important abnormalities [1–4].

Compared to CT, on state-of-the-art scanners, MRI provides a superior characterisation of abnormal or inconclusive gynaecological findings. If scanner availability and patient’s conditions permit, the use of MRI is attractive for further investigation of acute female genital disorders without ionising radiation [5–7].

This pictorial essay aims to provide radiologists with an increased familiarity in recognition and characterisation of acute gynaecologic disorders on CT and MRI, in order to allow timely diagnosis and appropriate treatment. The previous first instalment reviewed the cross-sectional imaging appearances of corpus luteum and haemorrhagic ovarian cysts, gynaecologic haemoperitoneum from either ruptured corpus luteum and ectopic pregnancy and adnexal torsion. The present second instalment will discuss and present (with imaging examples) the main non-pregnancy-related uterine emergencies (including endometrial polyps, degenerated leiomyomas and uterine inversion) and the spectrum of pelvic inflammatory disease (PID) [8, 9].

## Uterine emergencies

Abnormal genital bleeding represents the characteristic and most common manifestation of acute uterine disorders and is one of the most common presentations in the emergency gynaecology unit as it accounts for approximately one-third of urgent gynaecologic visits. Causes encompass a wide spectrum of systemic and endocrine disorders, benign conditions (infection and focal abnormalities of the uterine cavity) and malignancies [10].

In reproductive-age women, when pregnancy is ruled out, clinical examination is required to search for vaginal and cervical abnormalities underlying acute bleeding. Additional imaging is required to detect or exclude abnormalities such as endometrial polyps or submucosal fibroids. Endometrial cancer is rarely encountered in pre-menopausal women, very exceptionally under 40 years of age. Dysfunctional uterine bleeding (idiopathic menorrhagia) represents a diagnosis of exclusion, in the absence of a recognizable pelvic pathology [11].

Transvaginal US remains the initial modality of choice in the evaluation of endometrial diseases, but the use of MRI to clarify abnormal or suspicious US findings before hysteroscopy is increasing, as it provides superior detection and characterisation of abnormalities in the uterine cavity [12].

### Endometrial polyps

Endometrial polyps (EP) represent a localised overgrowth of endometrial glands with a central stroma composed of fibrous tissue or smooth muscle. Sometimes asymptomatic, EP easily cause either menometrorrhagia in pre-menopausal women or post-menopausal bleeding. The prevalence ranges from 7.8% to 34.9% and increases with age; tamoxifen is a well-established risk factor [13, 14].

**Fig. 1:**
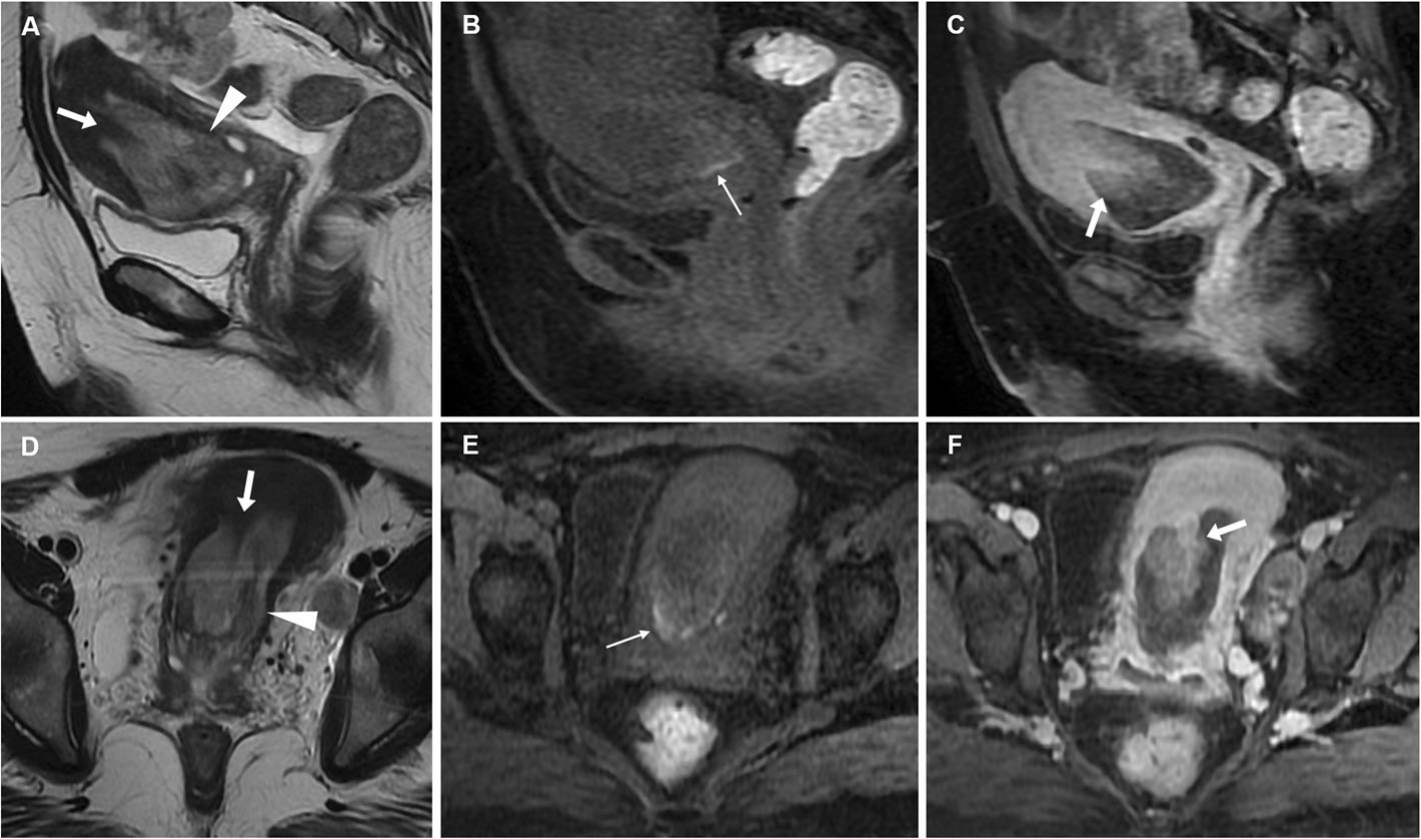
Endometrial polyp in a 44-year-old woman with acute uterine bleeding and associated bilateral ovarian neoplasm. Sagittal (**a**) and oblique-coronal (**d**) T2-weighted images show a huge polypoid mass (arrowheads) arising from the uterine fundus that fills the endometrial cavity and protrudes into the cervical canal. Note the hypointense stromal axis of the polyp (arrows). Corresponding sagittal (**b**) and oblique-coronal (**e**) precontrast fat-suppressed T1-weighted images show hyperintense foci on the polyp edge (thin arrows), representing haemorrhage. On gadolinium-enhanced sagittal (**c**) and oblique-coronal (**f**) fat-suppressed T1-weighted images, the implant base (stromal axis) of the polyp shows intense enhancement (arrows). The patient underwent bilateral uterine artery embolisation in order to control bleeding

On T2-weighted MR images, EP appear as low signal intensity sessile or pedunculated masses projecting into the endometrial cavity, surrounded by high signal intensity fluid and endometrium. Furthermore, a fibrous core (low signal intensity stripe or centre) and intratumoural cysts (discrete, smooth-walled cystic structures of high signal intensity) may be identified within the mass. Cystic spaces correspond to dilated glands but are nonspecific as they may be present within EP, hyperplasia or cancer. On T1-weighted images, EP generally have isointense signal compared to the endometrium, and may become haemorrhagic after ulceration or infarction leading to the appearance of hyperintense region best appreciated using precontrast fat saturation. After gadolinium contrast, EP may show either early persistent or gradually increasing enhancement, equal to or greater than that of outer myometrium (Fig. 1) [10, 15, 16].

Regarding management, hysteroscopic polypectomy is the gold standard for both diagnosis and treatment. Conservative management is reasonable in the case of small polyps in asymptomatic patients [17].

**Fig. 2:**
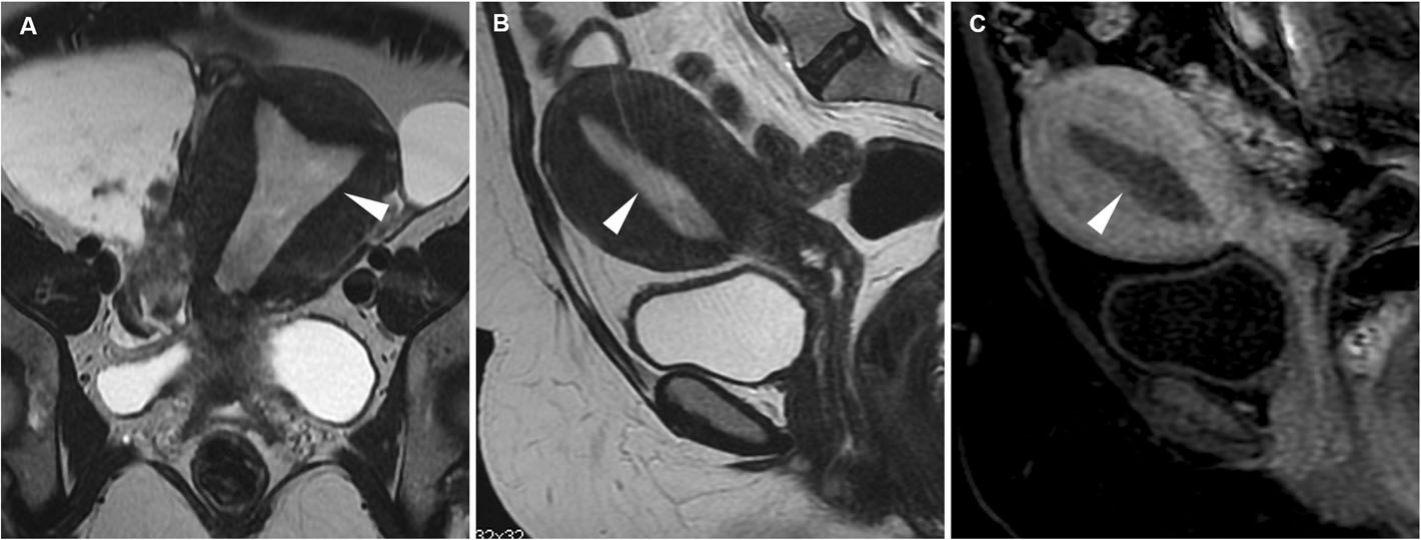
Endometrial hyperplasia in a 43-year-old woman with acute abnormal uterine bleeding. Oblique-coronal (**a**), sagittal (**b**) T2-weighted images and gadolinium-enhanced sagittal (**c**) fat-suppressed T1-weighted image show marked endometrial thickening (arrowheads) without macroscopic signs of myometrial invasion. Hysteroscopy with endometrial biopsy demonstrated simple endometrial hyperplasia without atypia

The key differential diagnosis of post-menopausal bleeding is endometrial carcinoma, which usually shows intermediate signal intensity (hyperintense compared with the myometrium) on T2-weighted images [18]. Identification of the central fibrous core and of intratumoural cysts favour the diagnosis of EP. Conversely, myometrial invasion, necrosis, a lower enhancement compared to the adjacent myometrium and irregular internal “cystic” areas suggest carcinoma. Endometrial hyperplasia is a premalignant condition that does not have a characteristic imaging appearance (Fig. 2); therefore, any focal endometrial thickening warrants hysteroscopy and biopsy, especially in post-menopausal women [10, 15, 16].

In post-menopausal women, the normal endometrium measures a mean 4 mm (range 1–12 mm) thickness [19], and endometrial thickness should be regarded as abnormal if ≥ 5 mm or > 9 mm, respectively, in presence or absence or genital bleeding [10].

In reproductive age, the normal endometrial thickness varies according to the menstrual cycle phases. Shitano et al. prospectively investigated the MRI appearance of normal endometrium in 32 healthy pre-menopausal women in follicular phase (FP) and luteal phase (LP). The maximum thickness of the normal endometrium, measured on sagittal T2-weighted images, in FP (mean 6.5 mm, range 2.1–14 mm) was significantly lower than that in LP (mean 10.4 mm, range 3.9–20.4 mm). Furthermore, on T2-weighted sequences, the signal intensity of the normal endometrium in FP was significantly higher than that in LP [20].

### Uterine fibroids

The most common gynaecological mass, fibroids (leiomyomas) are benign tumours composed of smooth muscle cells and fibrous connective tissues that develop in almost 20–30% of reproductive-age women. In non-complicated fibroids, CT findings include uterine enlargement with lobulated contours, deformity of the endometrial cavity, and presence of focal masses that vary from exophytic or subserosal to intramural to submucosal. When present, coarse calcification is quite specific. Contrast enhancement is variable, so that fibroids may appear isodense, hypodense or hyperdense relative to the myometrium. MRI is the most accurate modality to detect, localise and characterise leiomyomas, which are typically T2-hypointense compared to the surrounding myometrium and of intermediate signal intensity on T1-weighted images [21].

Acute pain develops in up to 30% of patients, secondary to either acute degeneration or torsion of an exophytic or submucosal pedunculated leiomyoma; in the latter case, vaginal bleeding coexists. Degeneration of leiomyomas results from the volumetric increase that outgrows vascular supply and may take different forms (hyaline, myxoid, cystic and haemorrhagic) according to the rapidity of development. The most common form is the hyaline one, with a deposit of collagen fibres. Among the different types of degeneration, the haemorrhagic one is most likely to cause acute pelvic pain. Haemorrhagic (“red”) degeneration often occurs during pregnancy or in association with the use of oral contraceptives and consists of haemorrhagic infarction of the leiomyoma secondary to venous thrombosis at the periphery of the lesion [21].

**Fig. 3:**
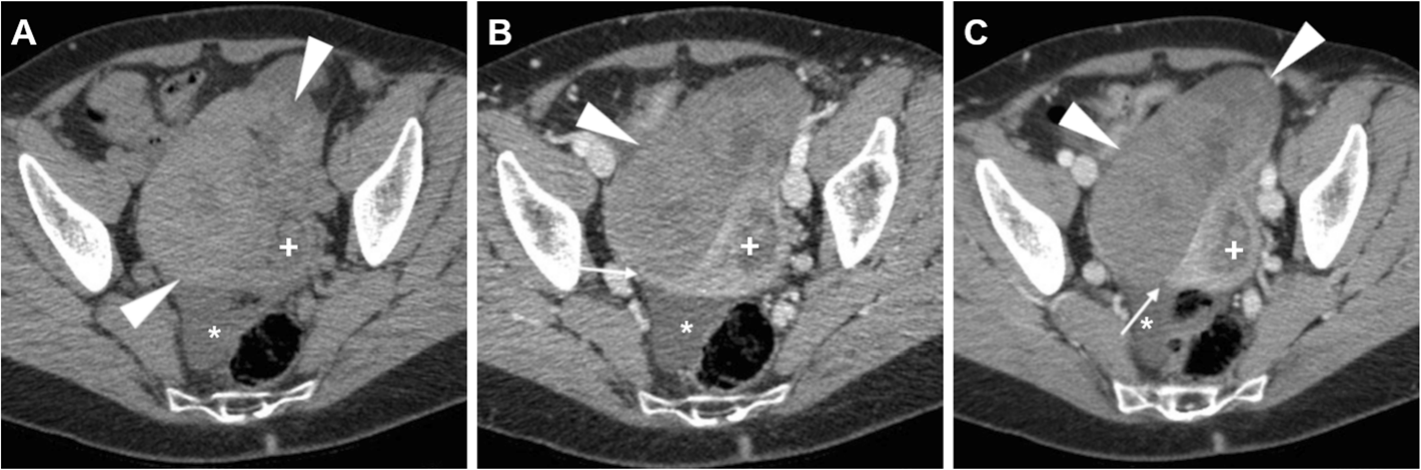
Surgically proven torsed uterine fibroma in a 64-year-old woman. Precontrast (**a**), arterial (**b**) and venous (**c**) phase CT images show a large, ovoid-shaped heterogeneous, mildly hyperattenuating and poorly enhancing mass (arrowheads) that displaces the uterus (plus sign) posterolaterally. Note the acute angle (thin arrow) indicating subserosal origin of the mass, and minimal effusion (asterisk) in the peritoneal cul-de-sac

**Fig. 4:**
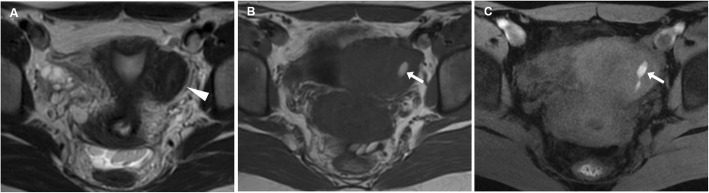
Leiomyoma with haemorrhagic degeneration in a 25-year-old woman suffering from acute pelvic pain. Oblique-coronal T2-weighted image (**a**) shows a subserosal leiomyoma, with predominant low signal intensity, arising from the left lateral wall of the uterus (arrowhead). On oblique-axial T1-weighted (**b**) and fat-suppressed T1-weighted (**c**) images, the mass demonstrates a central region of hyperintense signal (arrows) corresponding to haemorrhagic degeneration

**Fig. 5:**
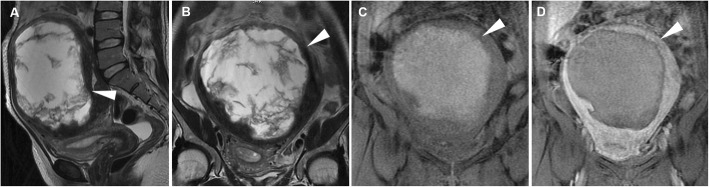
Leiomyoma with haemorrhagic degeneration in a 37-year-old woman with a palpable abdominal mass, pelvic pain and fever. Sagittal (**a**) and oblique-coronal (**b**) T2-weighted images show a huge subserosal leiomyoma arising from the posterior wall of the uterus, with heterogeneous signal intensity (arrowheads). On oblique-coronal fat-suppressed T1-weighted image (**c**), the mass demonstrates hyperintense signal (arrowhead) consistent with haemorrhagic degeneration, without contrast enhancement after gadolinium administration (**d**)

At CT, haemorrhage and loss of contrast enhancement within a pedunculated, subserosal or intramural fibroid with a development of a cystic-like appearance may suggest the possibility of degeneration or infarction (Fig. 3). At MRI, haemorrhagic leiomyomas display diffuse or peripheral high signal intensity on T1-weighted images, reflecting the effect of methaemoglobin. On T2-weighted sequences, they show variable signal intensity with peripheral hypointensity secondary to haemosiderin formation. After gadolinium contrast agent administration, the lack of enhancement reflects interrupted blood supply (Figs. 4 and 5) [2, 9].

The most challenging differential diagnosis of benign degenerating leiomyomas is from uterine leiomyosarcomas that may exhibit different MR imaging patterns: (a) lobulated mass with high signal intensity on T2-weighted sequences, (b) well-marginated mass with low signal (similar to that of a leiomyoma), or (c) mass with extensive invasion and infiltrative margins. The suspicion of leiomyosarcoma may also be raised by the identification of haemorrhage and coagulative necrosis. The latter can present as areas of slightly high signal intensity on T1-weighted sequences and heterogeneous signal intensity on T2-weighted sequences [22]. In the differential diagnosis between leiomyomas and leiomyosarcomas, intravenous contrast agent administration is mandatory to identify solid components of leiomyosarcomas [23]. Lakhman et al. identified four qualitative MR features (nodular borders, haemorrhage, “T2 dark” areas and central unenhanced areas) strongly associated with leiomyosarcomas. In particular, the combination of at least three of these MR features may distinguish leiomyosarcomas from atypical leiomyomas with a specificity of > 95% [24].

As for the role of DWI, restricted diffusion may represent a pitfall since it is observable in benign cellular leiomyomas as well as in leiomyosarcomas, although some authors demonstrated significantly lower mean ADCs in leiomyosarcomas than in degenerated leiomyomas [25]. Although invasion, necrosis and above all rapid growth are suggestive of malignancy, the differentiation between benign degenerating leiomyomas and leiomyosarcomas still remains particularly challenging and the final diagnosis is often established histologically [22].

In patients with symptomatic uterine leiomyomas, embolisation is recognised as a minimally invasive uterine-sparing treatment option. Although embolisation is generally safe, post-procedural pain may occur: pain may range from mild to severe, is more frequent during the first week after embolisation and forms the “post-embolisation syndrome” along with fever, loss of appetite, nausea and malaise. Post-procedural readmission is needed in about 10% of cases [26, 27]. Severe pain is partly explained by myometrial ischaemia and is correlated with the percentage and volume of ischaemic tissue [28].

**Fig. 6:**
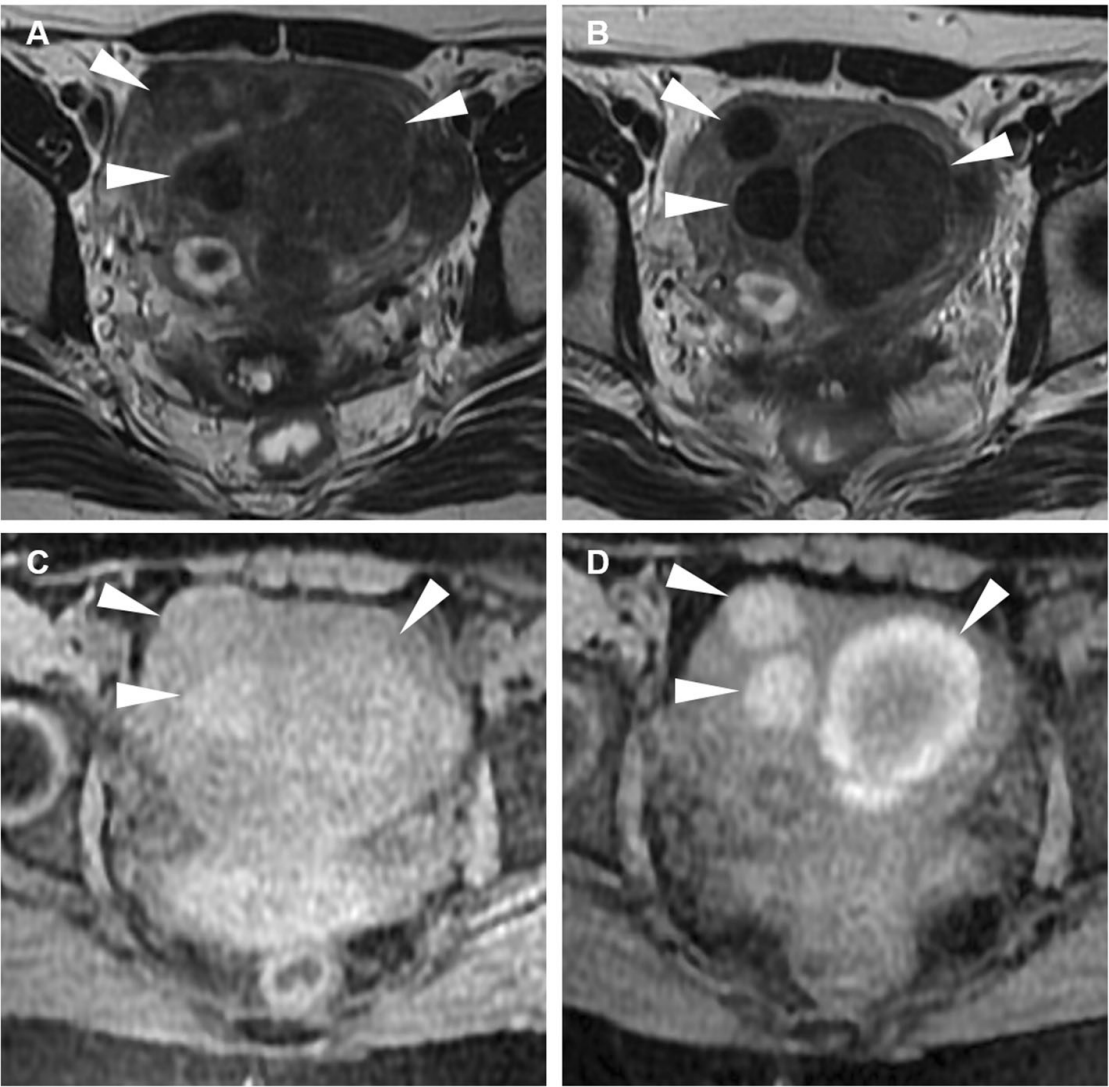
Multiple leiomyomas in a 48-year-old woman with pelvic pain and fever after uterine fibroid embolisation (UFE). Pre-treatment oblique-coronal T2-weighted (**a**) and fat-suppressed T1-weighted (**c**) images show multiple intramural leiomyomas, with respectively low and intermediate signal intensity (arrowheads). After bilateral UFE, on oblique-coronal T2-weighted (**b**) and fat-suppressed T1-weighted (**d**) images, the leiomyomas (arrowheads) show low T2 signal intensity and peripheral or homogeneous high signal intensity on fat-suppressed T1-weighted image reflecting internal haemorrhagic necrosis

Contrast-enhanced MRI is the imaging method of choice to evaluate post-embolisation appearances of fibroids. At follow-up, treated leiomyomas may show increased signal intensity on T1-weighted images, due to the shortening effect of methaemoglobin, and variable signal intensity on T2-weighted images, a finding consistent with haemorrhagic infarction induced by the procedure (Fig. 6). On contrast-enhanced T1-weighted images, the lack of enhancement within fibroids is indicative of successful complete infarction without residual viable tissue [29, 30]. Since imaging appearances of treated leiomyomas can be very similar to that of leiomyomas with red degeneration and, to some extent, of leiomyosarcomas with coagulative necrosis, particular attention should be paid in the anamnesis of patients with pelvic pain, with thorough questioning regarding possible previous interventional procedures.

### Uterine inversion

Uterine inversion (UI) refers to inside-out overturning and protrusion of the uterine fundus downwards up to or through the cervix that may occur either as an acute (within 24 h) life-threatening obstetric complication of mismanaged labour or in multiparous post-menopausal women from pulling effect of submucosal or pedunculated leiomyomas attached to the fundus; in the latter case, symptoms include pelvic tenderness or pain, vaginal discharge and irregular uterine bleeding. Physically, the vagina is occupied by the inverted uterus, but the clinical diagnosis is challenging without a high index of suspicion. Imaging is crucial to avoid misinterpretation as cervical tumour and to obviate biopsy which may cause profuse bleeding [31, 32].

**Fig. 7:**
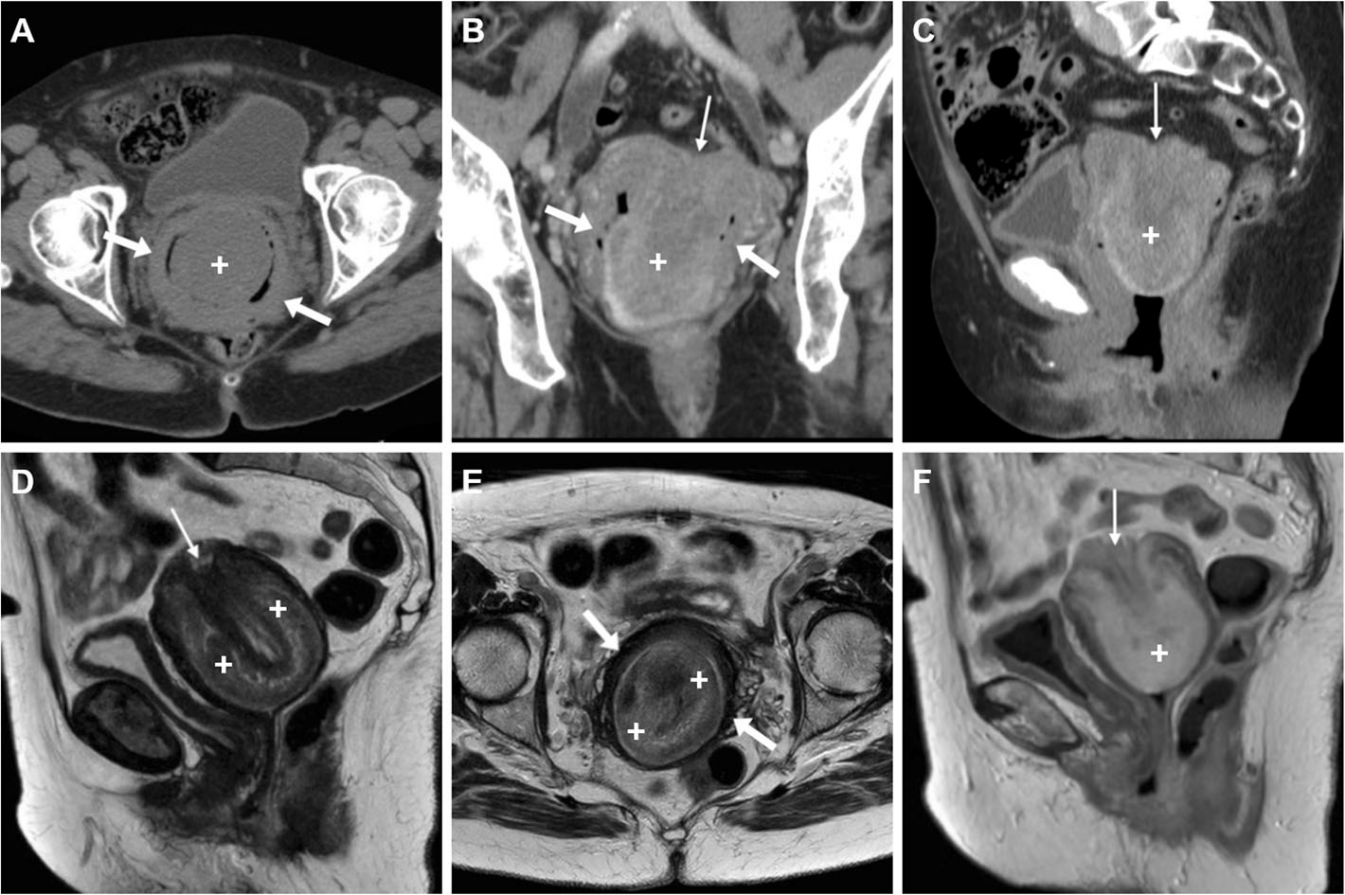
Surgically confirmed, spontaneous uterine inversion without mass lesions in a 79-year-old woman experiencing muco-haemorrhagic vaginal discharge. Precontrast (**a**) and contrast-enhanced (**b**, **c**) CT images showed a solid, mass-like enhancing structure (plus sign) surrounded by air coursing through the dilated uterine cervix. Loss of the normally convex uterine fundus (thin arrows) was noted. Physically, the upper vagina was occupied by the inverted uterus. Sagittal (**d**) and axial (**e**) T2-weighted MRI images confirmed the diagnosis by showing lost convexity and depression of the uterine fundus (thin arrows), U-shaped inverted uterus (plus sign) with preserved zonal anatomy for age and normal homogeneous enhancement on post-gadolinium T1-weighted image (**f**), which coursed downwards through the hypointense uterine cervix walls (thin arrows) [adapted from Open Access ref. no [33]]

Whereas the nature of the “mass” protruding into the vagina is difficult to identify at US, MRI generally clinches the diagnosis. The hallmark of UI on sagittal viewing is a U-shaped uterus with indentation and depression of the fundus, and a “bulls-eye” transverse configuration reflecting the zonal anatomy (Fig. 7d, f). Additionally, MRI may detect the possible presence of T2-hypointense submucosal fibroids or heterogeneously hyperintense mass-forming tumours. Albeit with limited contrast resolution, in acute settings, CT with adequate image reformation may also allow recognition of UI (Fig. 7a, c) [34].

Treatment of UI should consider the fertility and reproductive wishes of the patient, stage of inversion and underlying (benign or malignant) pathology. Whereas in puerperium manual repositioning is possible, post-menopausal UI requires abdominal or vaginal hysterectomy [35].

## Pelvic inflammatory disease

Acute PID currently affects nearly one million reproductive-age women in the USA, represents the single most common (almost 25%) cause of ED gynaecological visits and is increasingly encountered due to changing sexual habits. Risk factors include multiple sexual partners, high coital frequency and the presence of intrauterine contraceptive devices (IUCD). PID results from untreated ascending microbial infection from the vagina by sexually transmitted microorganisms such as *Chlamydia trachomatis*, *Neisseria gonorrhoeae*, *Mycoplasma genitalium* and gram-negative bacteria; mixed aerobic and anaerobic infections represent 30–40% of cases. Causative agents spread from the vagina to the cervix and uterine cavity, followed by the fallopian tubes and ovaries and even to the peritoneal cavity. Therefore, PID represents a spectrum of abnormalities encompassing cervicitis, endometritis, salpingitis, pyosalpinx and tubo-ovarian abscess (TOA) [36].

PID is often diagnosed clinically on the basis of fever, dull aching pelvic pain and cervical tenderness, vaginal mucopurulent discharge, leukocytosis and elevated C-reactive protein (CRP) levels. Most patients meeting these diagnostic criteria are treated empirically with broad-spectrum antibiotics. However, clinical manifestations are often unspecific. Therefore, cross-sectional imaging may be useful in patients with nonspecific presentation or inconclusive US, in those unresponsive to conventional therapy or with suspected complications such as abscesses requiring drainage. Furthermore, CT imaging is often requested in the ED to provide a differential diagnosis from other acute conditions such as urinary infection, appendicitis and diverticulitis [37, 38].

**Fig. 8:**
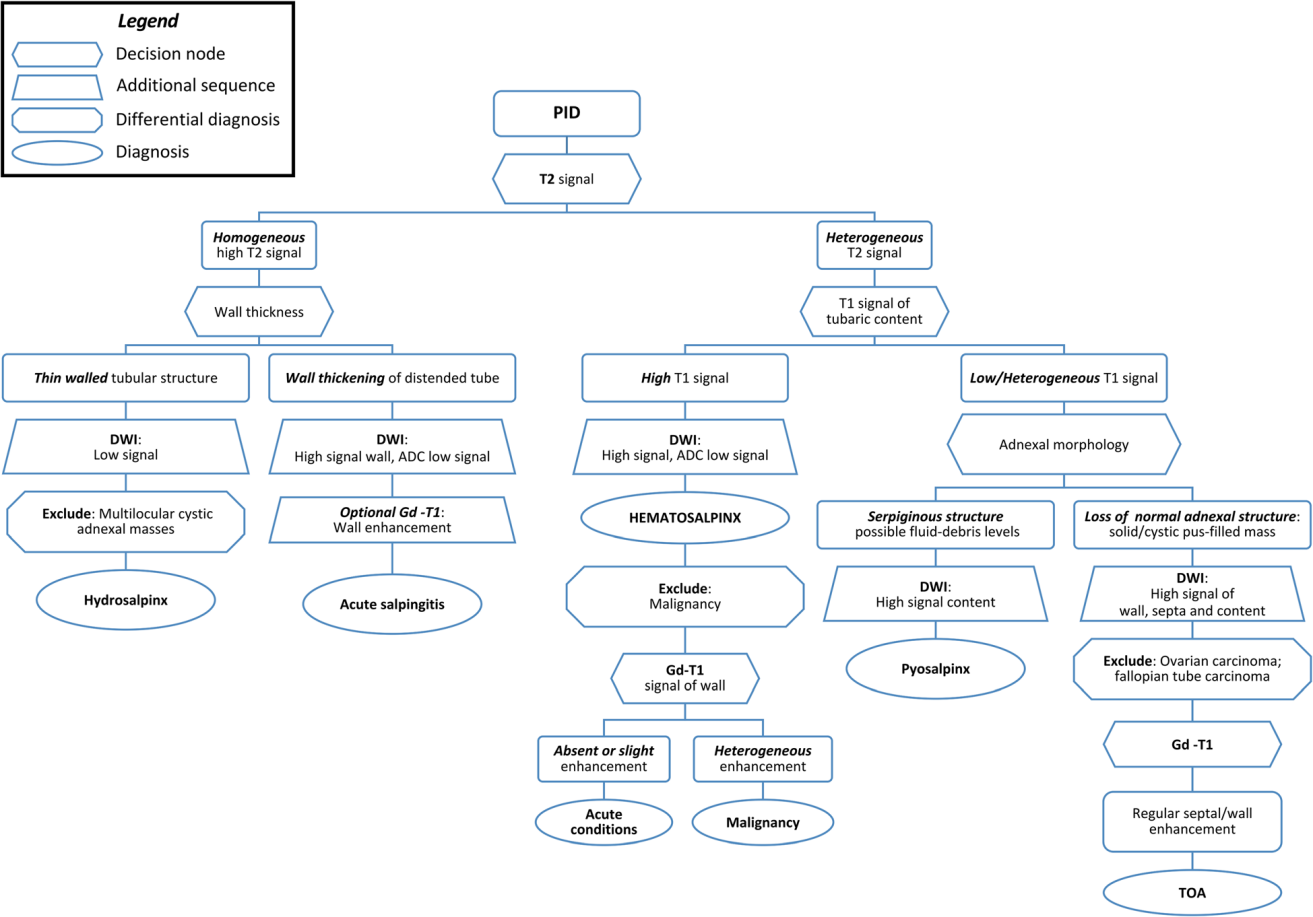
Flow-chart showing the MRI algorithm that proposes diagnostic steps for characterisation and differential diagnosis of pelvic inflammatory disease (PID) forms, according to signal intensity features, diffusion restriction, mural thickness and contrast enhancement

Tables 1 and 2 summarise the CT and MR imaging features of the PID spectrum, respectively. Additionally, a flow-chart describing an algorithm for MRI correct characterisation of PID forms and differential diagnosis is proposed in Fig. 8.

**Table 1:** CT findings of pelvic inflammatory disease (PID)

Tubo-ovarian disease	Morphology	CT	Contrast-enhanced CT
Hydrosalpinx	Fluid-filled thin-walled tubular structure	Tubal dilatation with fluid attenuation	No enhancement
Haematosalpinx	Dilated fallopian tube filled with blood products	Increased attenuation due to haemorrhagic content	Slight wall enhancement
Acute salpingitis	Swollen fallopian tube with intramural inflammation	Hypoattenuating fluid distension	Wall thickening and enhancement
Pyosalpinx	Bilateral, serpiginous or tubular structure with purulent content and possible fluid-debris levels	Fluid-debris levels, perivisceral fat stranding	Wall enhancement
Tubo-ovarian abscess	Loss of the normal adnexal structure, formation of a solid-cystic pus-filled inflammatory mass	Multilocular solid-cystic mass, low attenuation, perivisceral fat stranding	Wall and septal enhancement

**Table 2:** MR imaging findings of PID. *DWI* diffusion-weighted imaging, *ADC* apparent diffusion coefficient

Tubo-ovarian disease	Morphology	T2	T1	Gd–T1	DWI	ADC	Differential diagnoses
Hydrosalpinx	Fluid filled thin-walled tubular structure	High	Low	No enhancement	Low	No restriction	- Multilocular cystic adnexal masses
Haematosalpinx	Dilated fallopian tube filled with blood products	Intermediate with dark thick rim, possible “shading sign” in endometriosis	High	Slight enhancement of the tubaric wall	High	Restriction	- Endometriosis - Ectopic Pregnancy - Adnexal torsion - Trauma - Malignancy
Acute salpingitis	Swollen fallopian tube with intramural inflammation	Wall thickening of the distended tube	Low	Wall enhancement	High signal of the wall	Restriction of the wall	
Pyosalpinx	Bilateral, serpiginous or tubular structure with purulent content and possible fluid-debris levels	Iso-hyperintense	Variable	Enhancement of the thickened wall	High	Restriction	
Tubo-ovarian abscess	Loss of the normal adnexal structure, formation of a solid-cystic pus-filled inflammatory mass	Heterogeneous high signal	Heterogeneous low signal	Septal and thick rim mucosal enhancement	High signal of the wall, septa and purulent content	Restriction of the wall, septa and purulent content	- Ovarian carcinoma - Primary fallopian tube carcinoma

### Cervicitis and endometritis

**Fig. 9:**
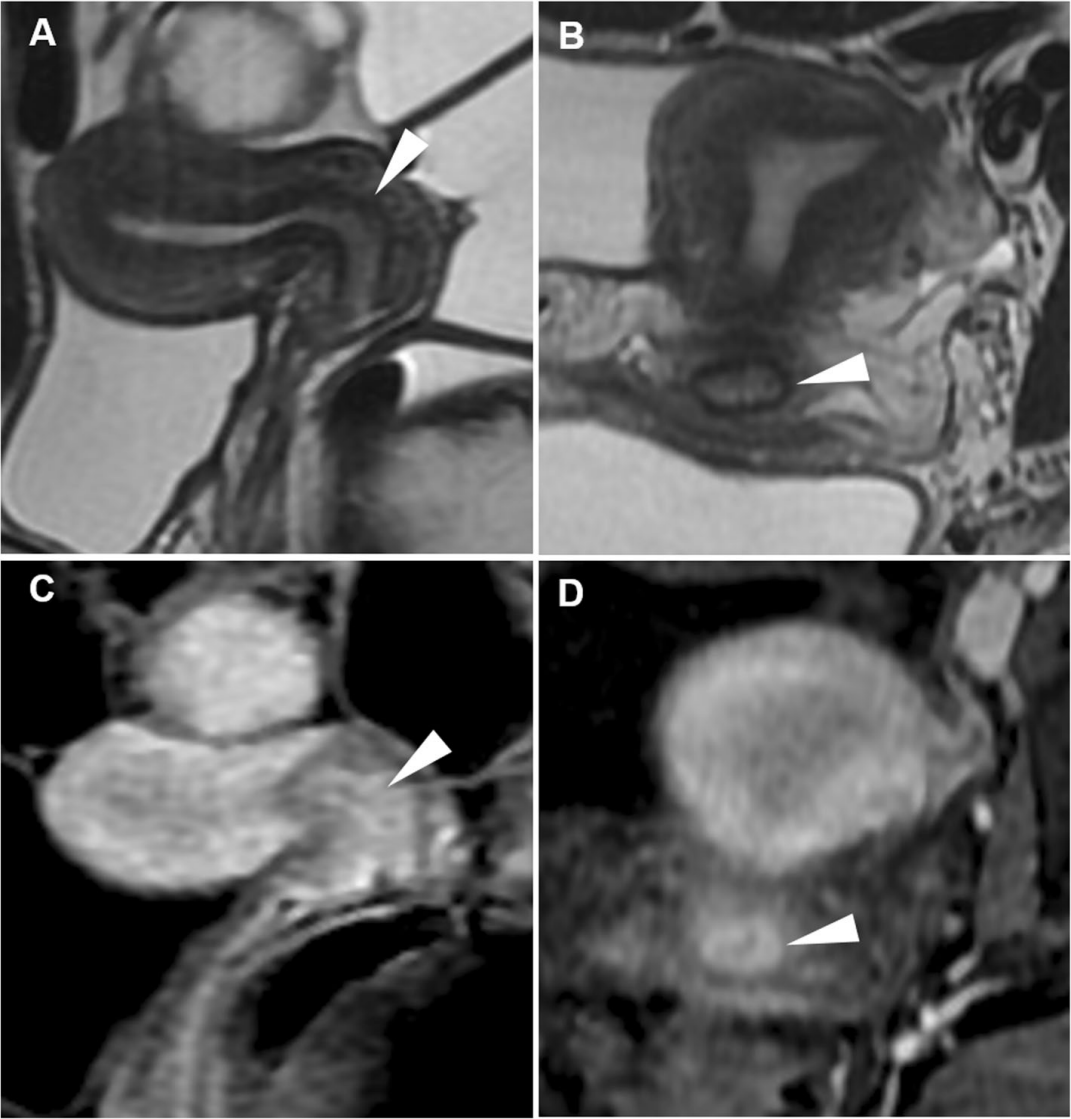
Cervicitis in a 24-year-old woman suffering from pelvic pain. Sagittal (**a**) and oblique-coronal (**b**) T2-weighted images show thickening of the mucosal layer at the cervical canal (arrowheads). The corresponding post-gadolinium sagittal (**c**) and oblique-coronal (**d**) fat-suppressed T1-weighted images show prominent enhancement along the cervical canal (arrowheads)

Cervicitis and endometritis represent early manifestations of PID and are often subclinical; when present, symptoms include pelvic pain, fever, vaginal discharge and menstrual abnormalities. In the former, MRI may demonstrate an enlarged uterine cervix with a prominent enhancement of the endocervical canal reflecting inflammation and hyperaemia (Fig. 9). Free pelvic effusion may be associated [39, 40].

**Fig. 10:**
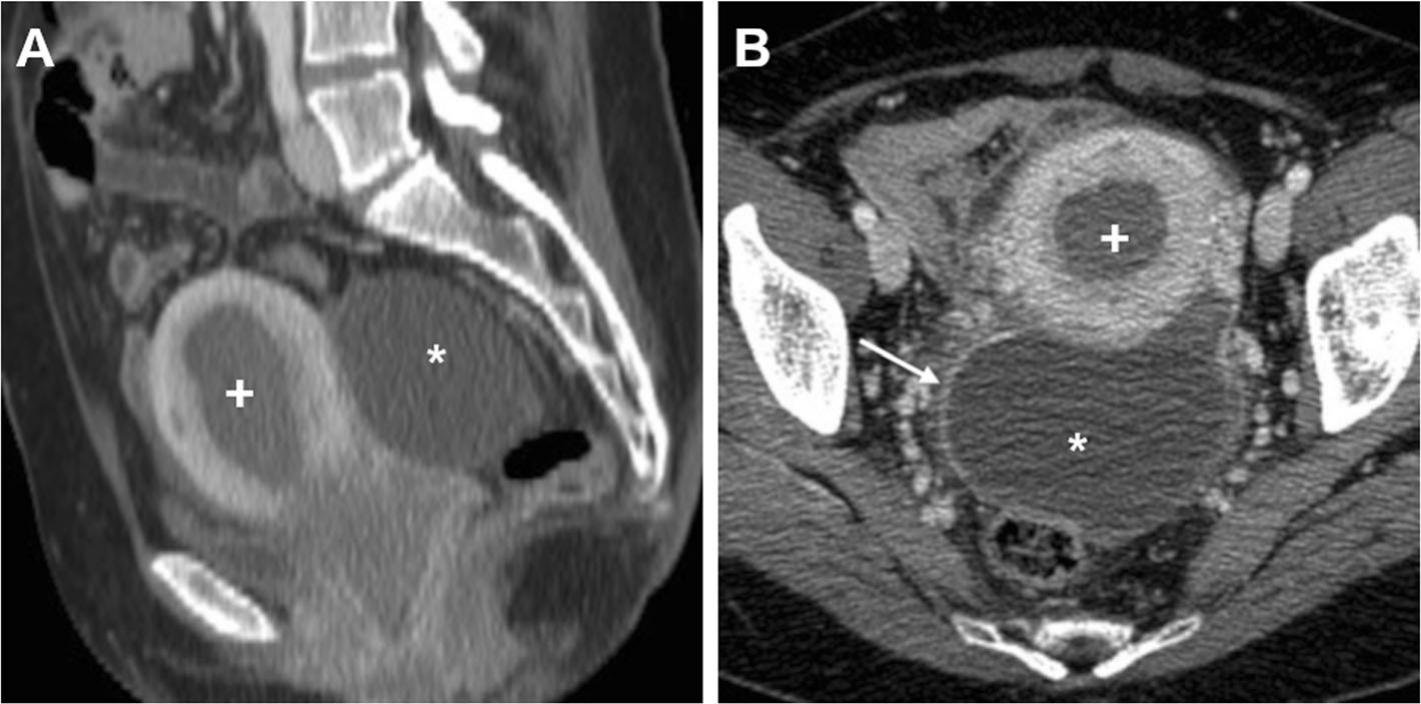
Haematometra and endometritis at CT. Sagittal (**a**) and axial (**b**) postcontrast CT images show markedly dilated uterine cavity (plus sign) filled by heterogeneous fluid. Associated loculated effusion (asterisk) in the pelvic cul-de-sac with thin serosal hyperenhancement (thin arrow in **b**)

**Fig. 11:**
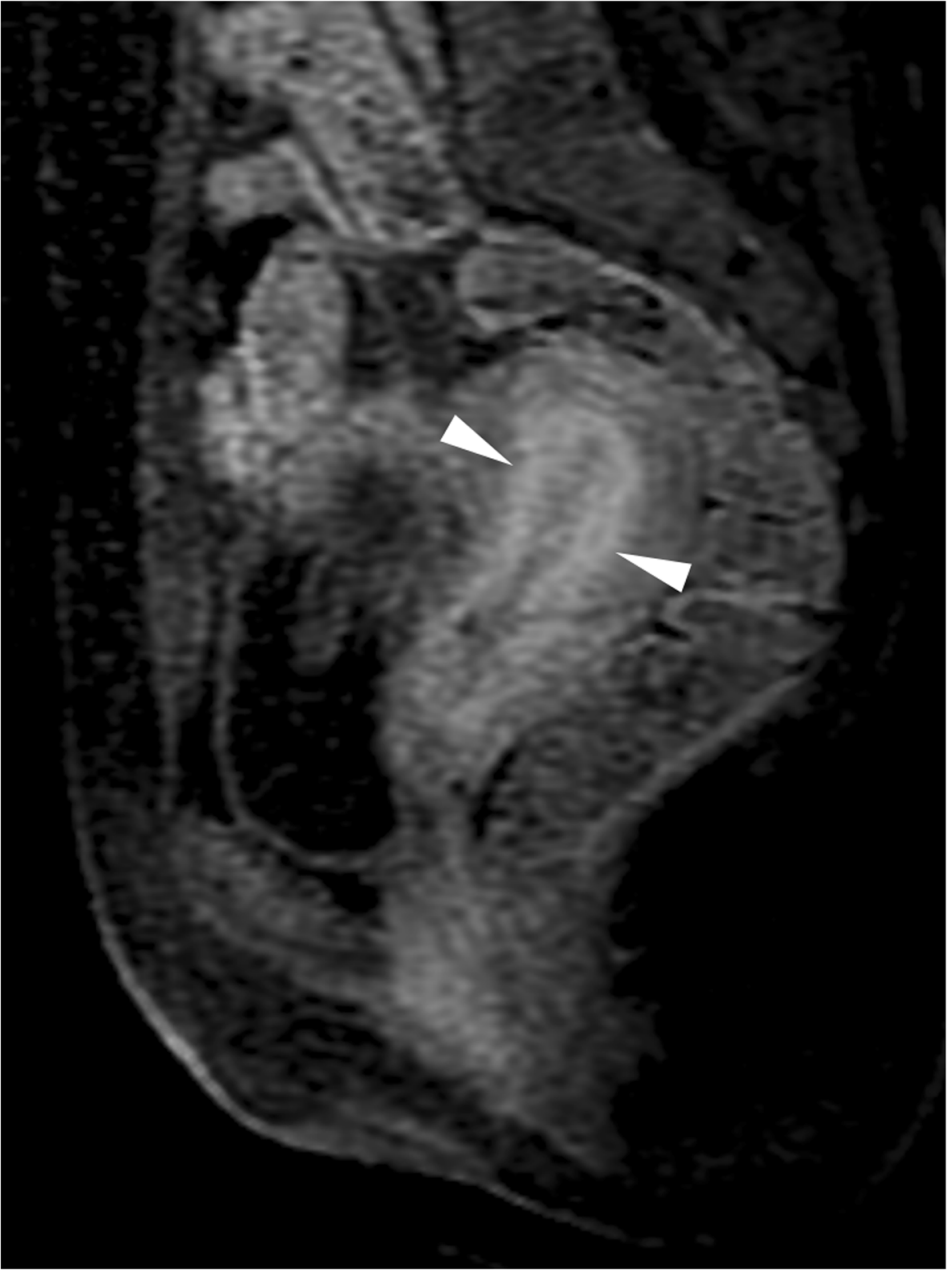
MRI appearance of endometritis in a 38-year-old woman with pelvic pain. Sagittal gadolinium-enhanced fat-suppressed T1-weighted image shows contrast enhancement of the endometrium and inner myometrial layer (arrowheads)

On the other hand, endometritis may be encountered during pregnancy, during the postpartum period, following invasive gynaecologic procedures or in the setting of PID. CT (Fig. 10) and MRI (Fig. 11) may demonstrate the presence of fluid in the endometrial cavity and abnormal endometrial enhancement. Additionally, the lack of a clear separation of the uterus from adnexal and parametrial tissue, with “indistinct uterine border”, may be detected [37].

### Pyosalpinx and differential diagnosis

**Fig. 12:**
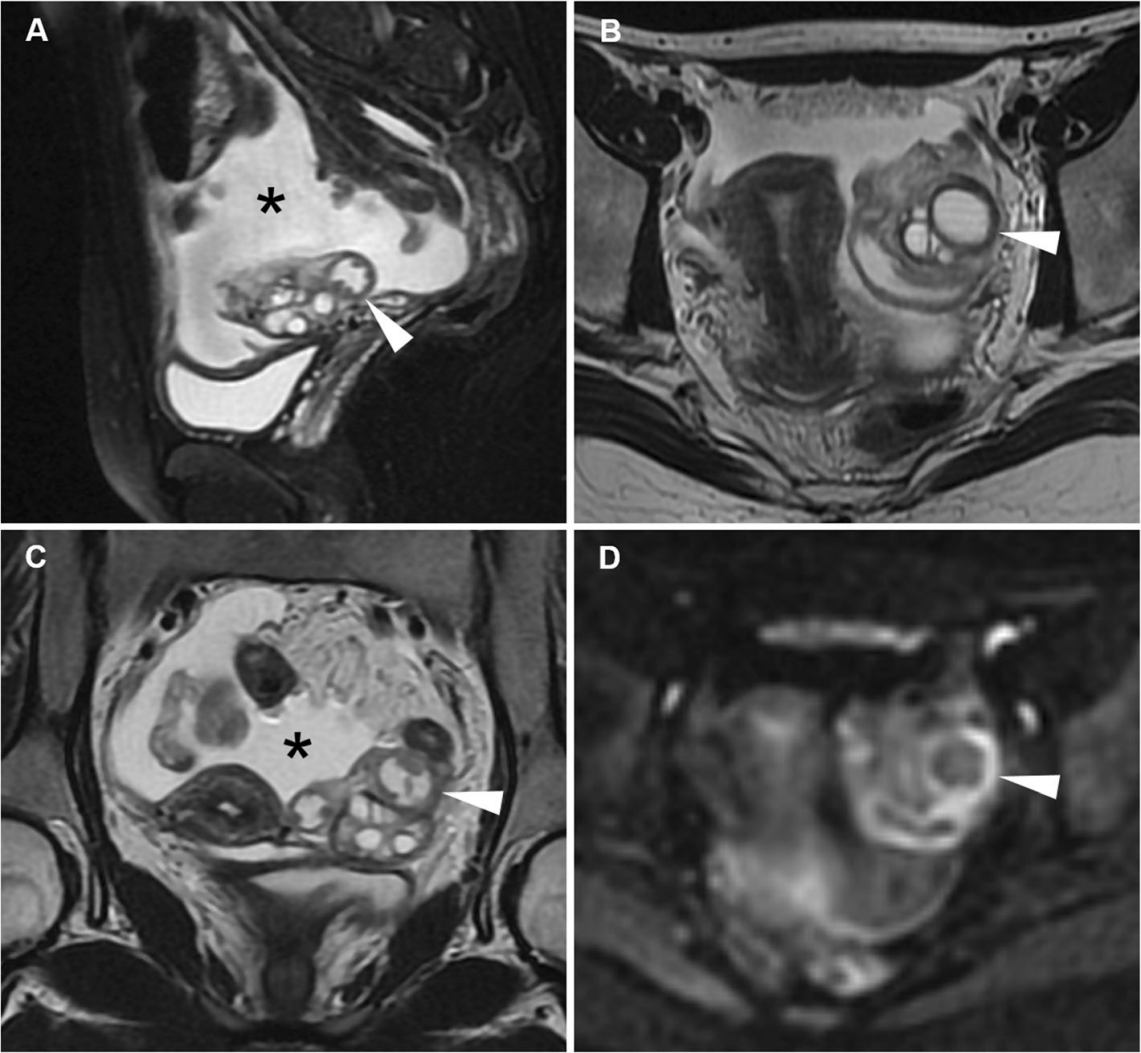
Acute salpingitis in a 25-year-old woman with pelvic pain and mild neutrophilic leukocytosis. Sagittal fat-suppressed (**a**), oblique-coronal (**b**) and oblique-axial (**c**) T2-weighted images show slightly dilated left fallopian tube with thickened walls (arrowheads). On axial diffusion-weighted imaging (DWI, *b* = 800 s/mm^2^) image (**d**), the tubal wall thickening demonstrates restricted diffusion (arrowheads), finding consistent with acute inflammation. Note pelvic peritoneal effusion (asterisk)

**Fig. 13:**
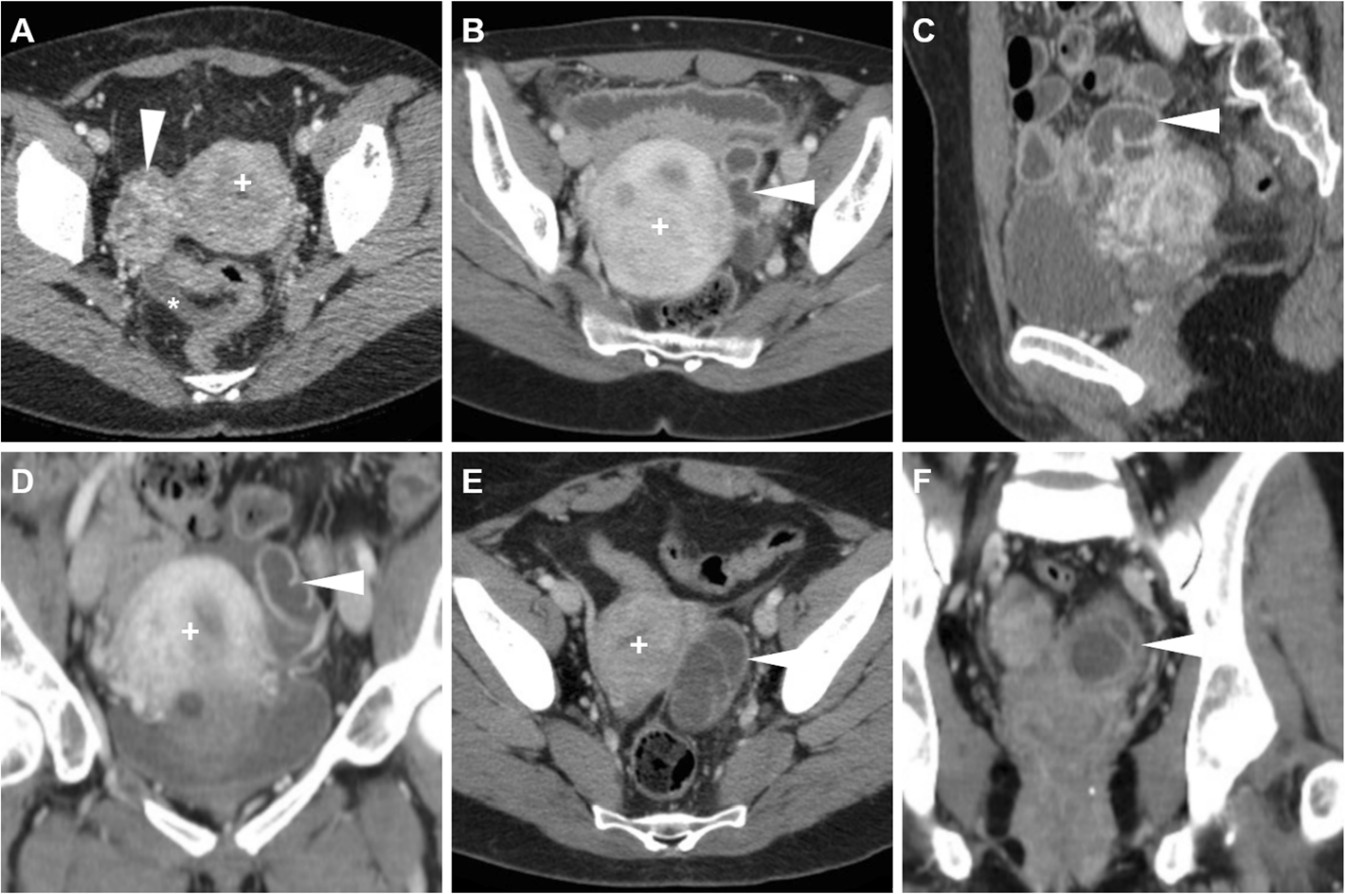
CT appearances in three different patients with a clinical diagnosis of PID. **a** Heterogeneously enhancing, unilaterally enlarged right ovary (arrowhead). Note uterus (plus sign), mild cul-de-sac effusion (asterisk). **b–d** Multiplanar images of tubular-shaped left salpingitis (arrowheads), distended with prominent mural enhancement. **e–f** Enlarged, septated left ovary (arrowheads) with thickened walls consistent with early tubo-ovarian abscess (TOA)

Salpingitis corresponds to a swollen fallopian tube with mural inflammation and thickening (Fig. 12). Conversely, pyosalpinx reflects superimposed infection with luminal distension by pus. At CT, multiplanar reformations are helpful to recognise the dilated fallopian tubes as serpentine or tubular juxta-uterine fluid-containing structures with a diameter over 5 mm, peripherally enhancing a thick wall and complex internal fluid (Fig. 13). Surrounding pelvic oedema, thickening of uterosacral ligaments, periuterine and adnexal fat stranding may be detected [1–4].

**Fig. 14:**
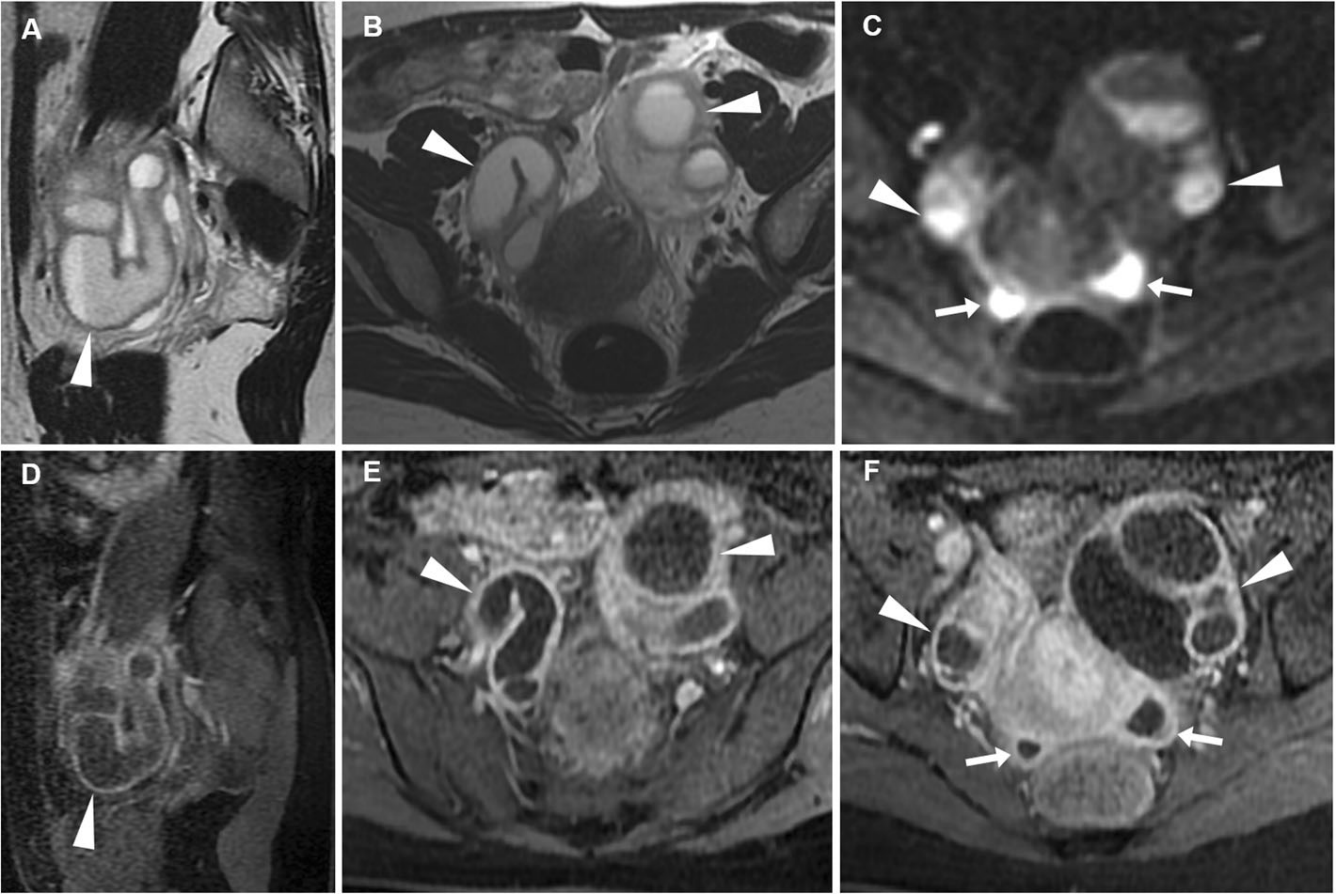
Acute pyosalpinx in the same patient as Fig. 11. Sagittal (**a**) and oblique-axial T2-weighted (**b**) images show distended fallopian tubes (arrowheads) with thickened walls and internal fluid-fluid levels. Axial DWI (*b* = 800 s/mm^2^) image (**c**) demonstrates high signal intensity indicating restricted diffusion of the tubal content (arrowheads) and of two fluid collections in the Douglas pouch (arrows), reflecting purulent content. Sagittal (**d**) and oblique-axial gadolinium-enhanced fat-suppressed (**e**, **f**) T1-weighted images show intense mural enhancement of the involved Fallopian tube (arrowheads) and of the fluid collections (arrows)

**Fig. 15:**
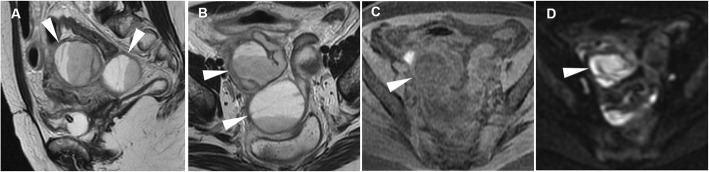
Acute pyosalpinx in a 44-year-old woman with a history of surgically treated pelvic endometriosis 5 years earlier, suffering from abdominal pain, fever and diarrhoea. Sagittal (**a**), axial (**b**) T2-weighted images and axial fat-suppressed T1-weighted (**c**) image show a distended right fallopian tube (arrowheads) with thickened walls and internal fluid-fluid levels. On axial DWI (*b* = 800 s/mm^2^) image (**d**), the involved fallopian tube (arrowhead) demonstrates high signal intensity reflecting restricted diffusion from purulent content

**Fig. 16:**
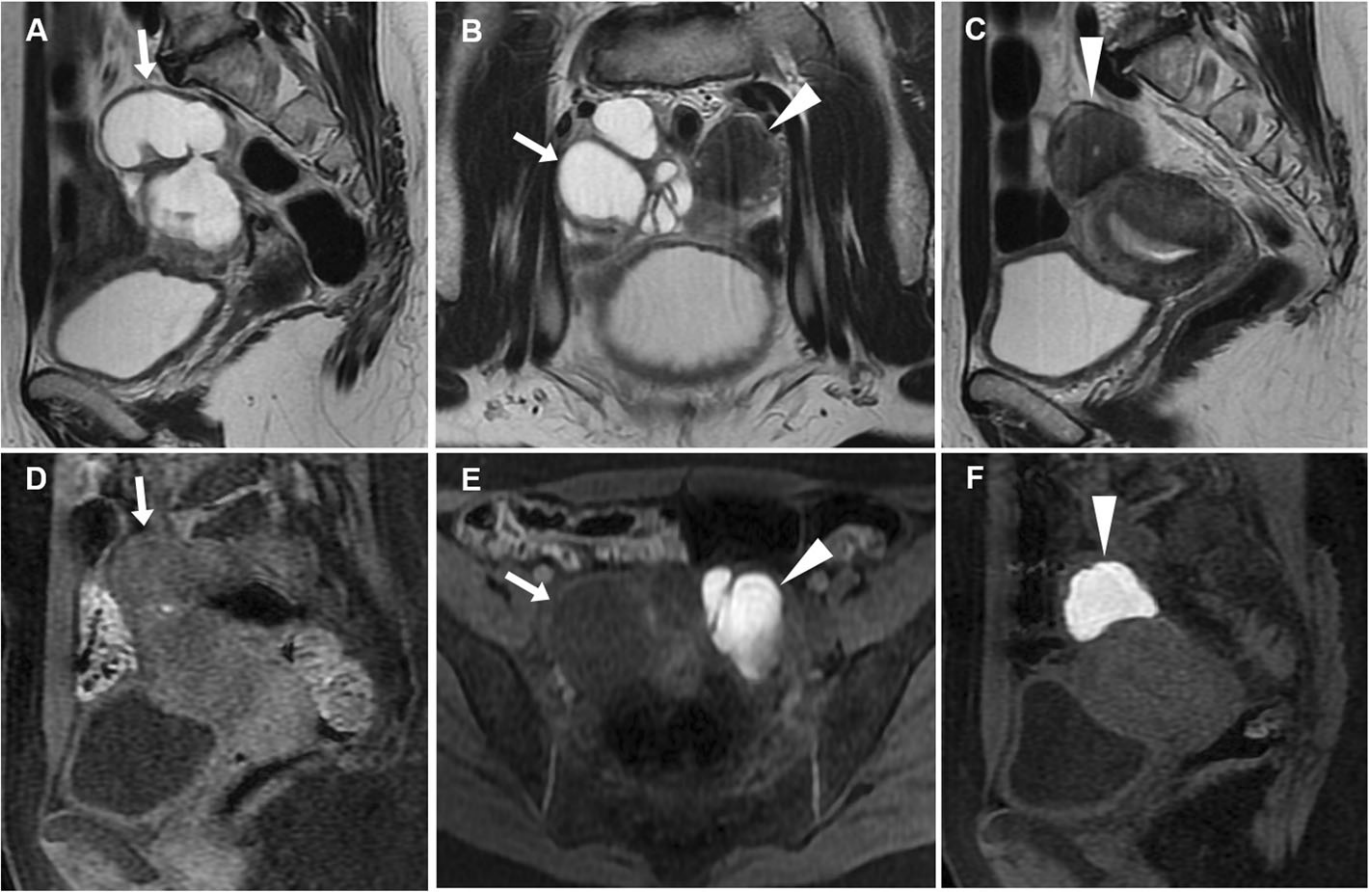
Acute onset of endometriosis in a 37-year-old woman with acute pelvic pain and fever for the last 4 days. Sagittal (**a**), oblique-coronal (**b**) T2-weighted images and sagittal (**d**), oblique-coronal (**e**) fat-suppressed T1-weighted images show a distended serpiginous right fallopian tube (arrows) with homogeneously high T2-weighted signal and low T1-signal intensity, consistent with hydrosalpinx. Additionally, oblique-coronal (**b**) and sagittal (**c**) T2-weighted images, oblique-coronal (**e**) and sagittal (**f**) fat-suppressed T1-weighted images demonstrate an endometriotic cyst of the left ovary (arrowheads) showing high T1 and low T2 signal (shading sign)

**Fig. 17:**
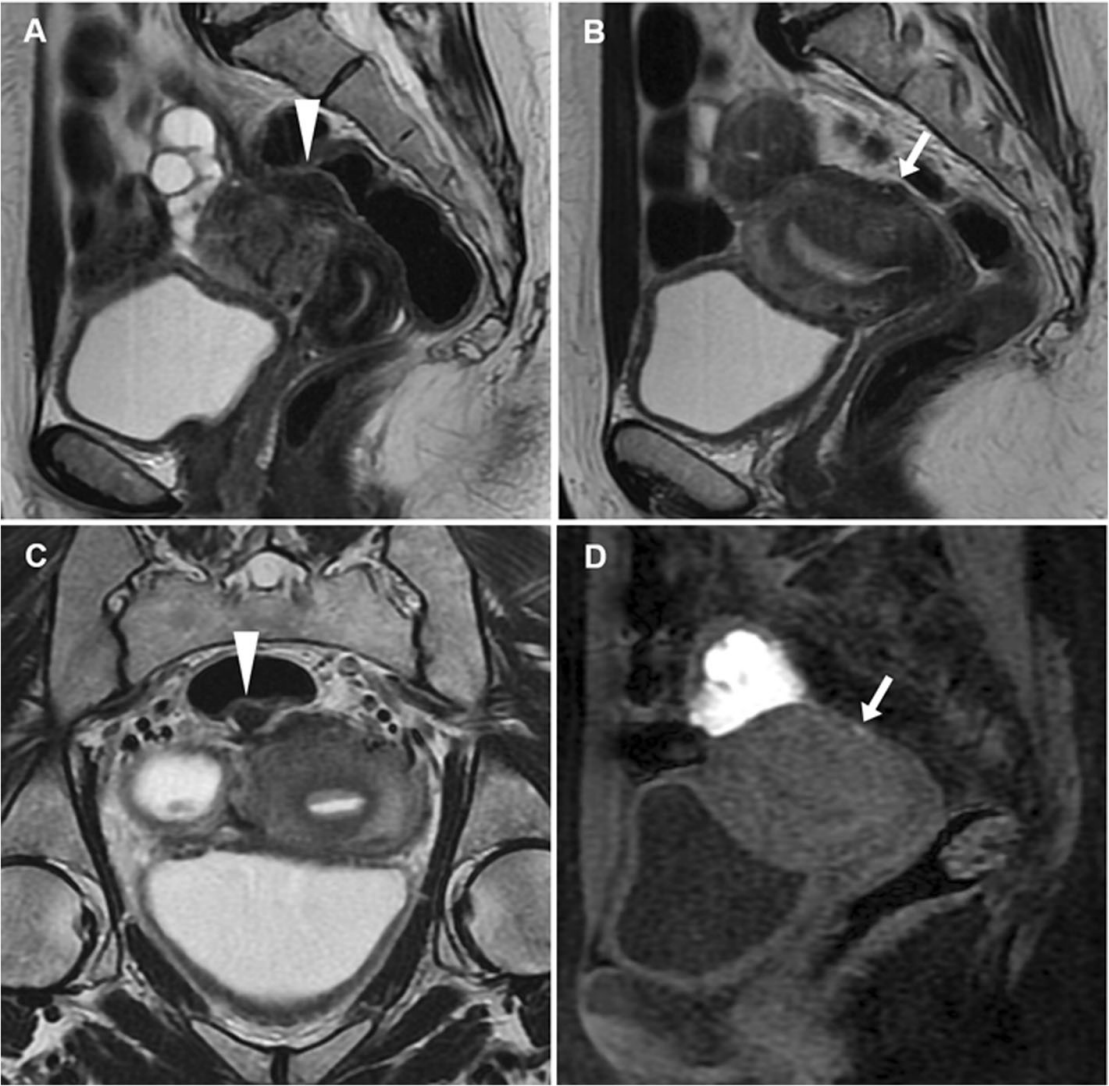
In the same patient as Fig. 16, sagittal (**a**) and oblique-axial (**c**) T2-weighted images show a hypointense endometriotic plaque (arrowheads) that infiltrates the muscular layer of the anterior rectal wall. Sagittal T2-weighted (**b**) and sagittal fat-suppressed T1-weighted (**d**) images show additional endometriotic implants of the uterine serosa (arrows) demonstrating low T2-weighted, high T1-weighted signal intensity

The strength of MRI relies on its ability to identify the ovary, to demonstrate the tubular nature of a mass, and to differentiate pyosalpinx from haematosalpinx on the basis of signal intensity of the tubal fluid. Tukeva et al. found MRI (sensitivity 95%, specificity 89% and 93% overall accuracy) to be more accurate than transvaginal US in the diagnosis of PID (corresponding values were 81%, 78%, and 80%) and to be useful in decreasing the need for diagnostic laparoscopy [39]. Compared to CT, MRI better identifies tubal wall thickening, inflammation and enhancement (Figs. 14 and 15) and therefore allows differentiation of pyosalpinx from hydrosalpinx (Figs. 16 and 17), in which the fallopian tube is not infected, but dilated secondary to obstruction of the ampullary segment; causes include prior episodes of PID, endometriosis, adhesions and prior ectopic pregnancy in descending order of frequency. At cross-sectional imaging, hydrosalpinx appears as a thin-walled tubular C- or S-shaped structure separated from the uterus and ovary, with typical fluid signal intensity; sometimes, the pathognomonic “cogwheel” cross-section appearance corresponding to thickened longitudinal folds may be identified. Interpreted in conjunction with conventional MRI sequences, diffusion-weighted imaging (DWI) helps in the characterisation of adnexal lesions by discriminating the nature of the tubal content: a water-like appearance (hypointense on T1-weighted images and strongly hyperintense on T2-weighted images) and unrestricted diffusion are suggestive of hydrosalpinx; variable or heterogeneous signal intensity on conventional MR sequences and restricted diffusion on DWI is a characteristic of pyosalpinx and TOA [41].

**Fig. 18:**
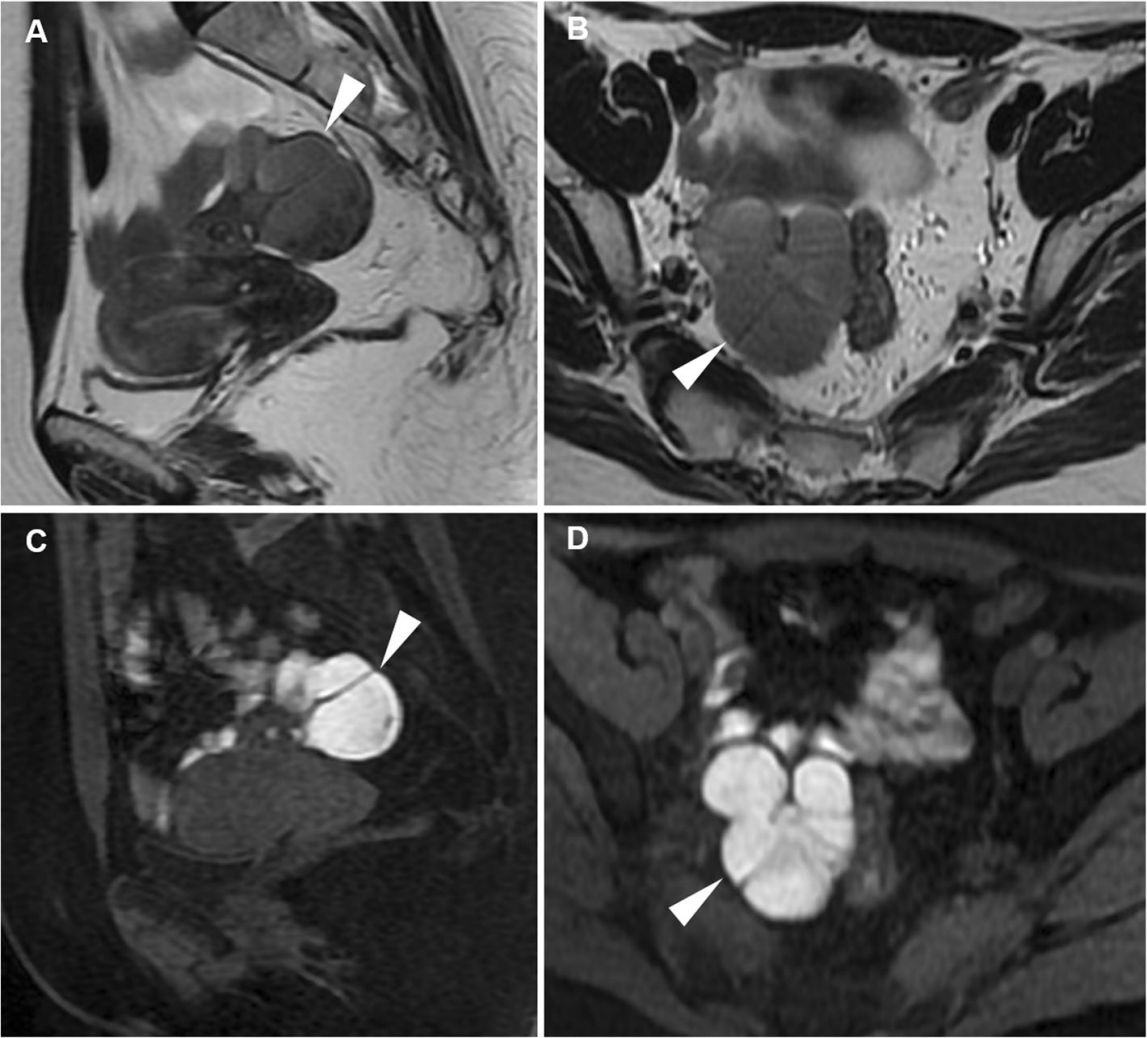
Haematosalpinx in a 34-year-old woman with endometriosis presenting with dysmenorrhea and pelvic pain. Sagittal (**a**) and oblique-coronal (**b**) T2-weighted images show a tortuous structure with homogeneously hypointense content in right adnexa (arrowheads). On sagittal (**c**) and oblique-coronal (**d**) fat-suppressed T1-weighted images the same structure (arrowheads) shows high signal intensity consistent with haemorrhagic content

Another differential diagnosis is haematosalpinx (Fig. 18), corresponding to dilatation of the fallopian tubes by blood products which may develop during EP, adnexal torsion, endometriosis, trauma and malignancy; the hallmark of haematosalpinx is hyperintense tubal content on fat-suppressed T1-weighted images, reflecting subacute blood [37, 38, 42, 43].

Posterior extension of the inflammatory process may cause thickening of the uterosacral ligaments, a common finding of PID, easily recognizable at both CT and MR [37]. Additionally, both CT and MRI may demonstrate reactive lymphadenopathy. Due to the course of drainage of the ovarian and salpingian lymphatic vessels along the gonadal veins, lymphadenopathy affects the paraaortic lymphatic chain at the level of the left renal hilum from the left side, and the paracaval and aortocaval chain from the right side [44].

### Tubo-ovarian abscess

TOA develops in up to 15% of patients and represents the full-blown manifestation of untreated PID, in which further progression of infection leads to complete destruction of the normal adnexal structure and formation of an inflammatory mass that encompasses both the fallopian tube and ovary. Making the diagnosis of TOA is important since it requires hospitalisation and sometimes image-guided or surgical drainage.

**Fig. 19:**
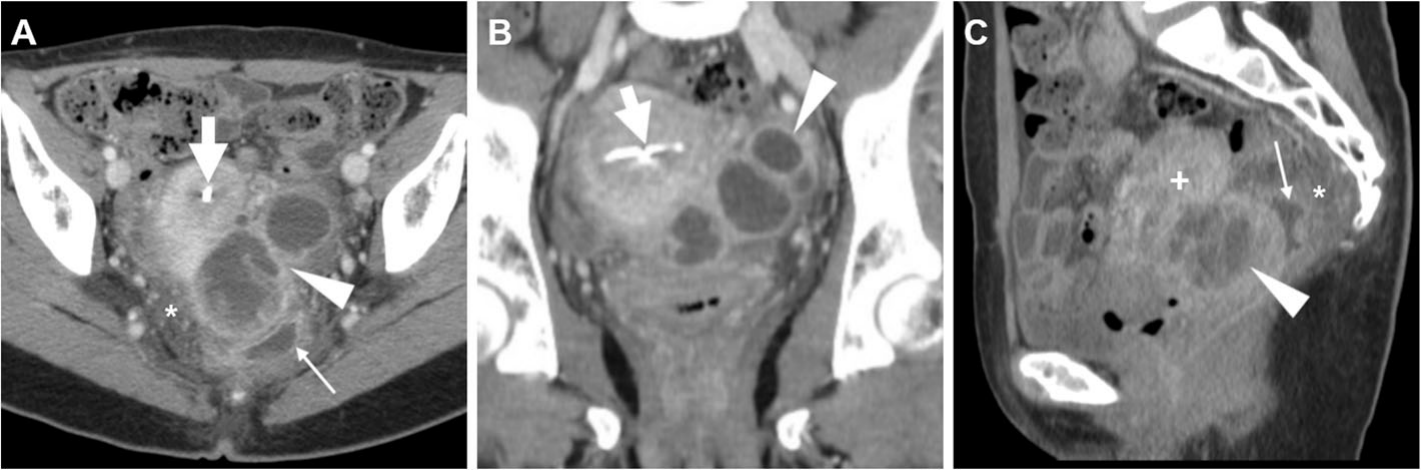
**a**–**c** Multiplanar CT images of a complex, septated left-sided TOA (arrowheads) in a 42-year-old woman, with characteristic irregular peripheral enhancement, that displaces the retroverted uterus (plus sign). Note metallic intrauterine contraceptive device (IUCD, thick arrows), minimal peritonitis of the cul-de-sac (thin arrows) and inflammatory stranding of the presacral fat (asterisk). The IUCD had to be removed

Sometimes bilateral, TOA shows up at CT (Figs. 13 and 19) as a mixed solid-cystic adnexal mass with low-attenuation, complex internal fluid and thickened and irregularly enhancing walls and septa. The ipsilateral mesosalpinx is frequently thickened and displaced anteriorly. The identification of dilated, pus-filled fallopian tube helps to distinguish a TOA from a complex neoplastic mass. Free peritoneal fluid, surrounding inflammation in the pelvis with stranding of the presacral and periovarian pelvic fat are generally present (Fig. 19) [1, 3, 4, 8, 43].

**Fig. 20:**
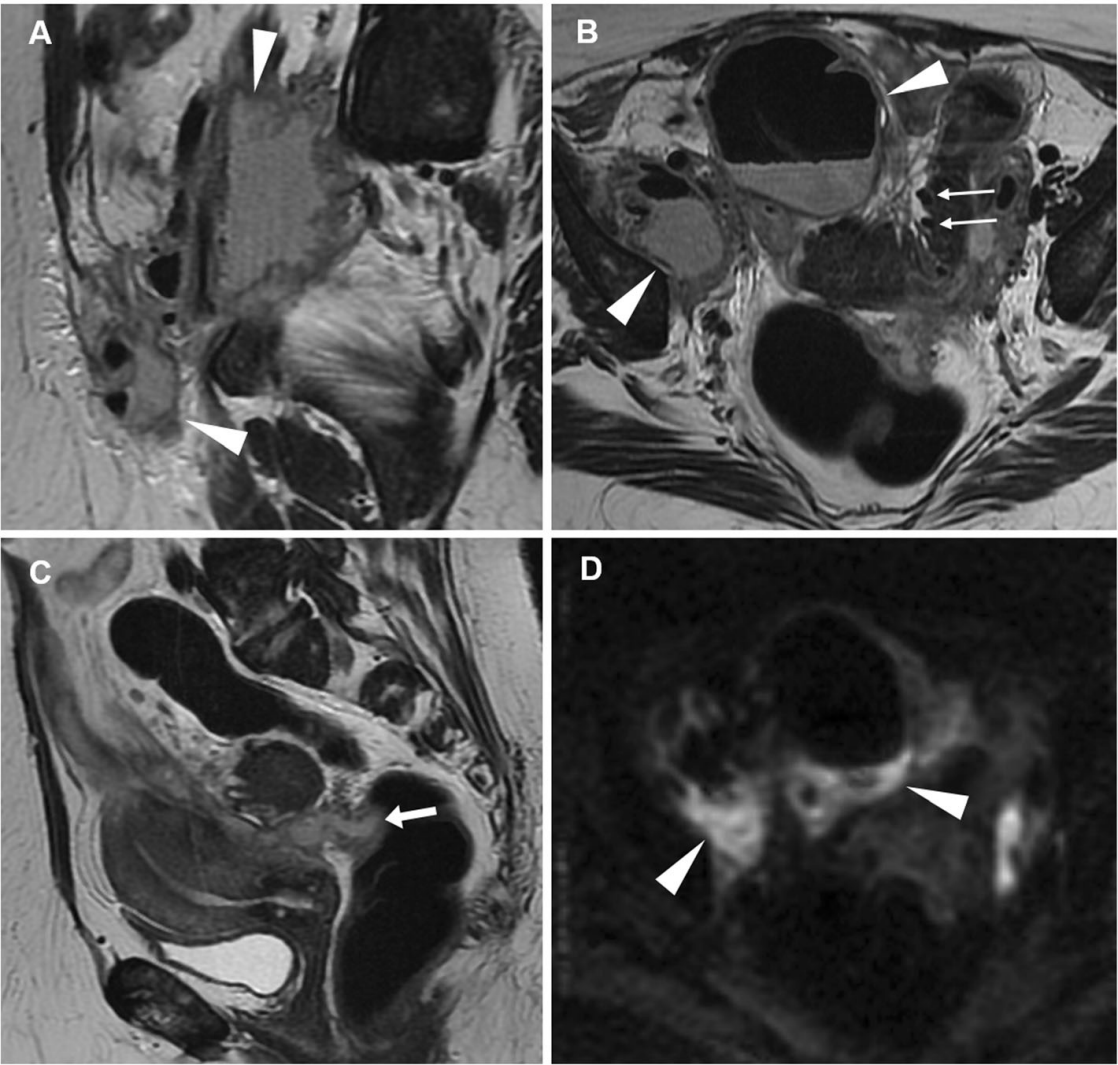
TOA in a 57-year-old woman with right lower quadrant pain, fever, leukocytosis and acute diverticulitis. Sagittal (**a**) and axial (**b**) T2-weighted images show heterogeneous, multilocular collections at the right adnexa (arrowheads), containing air-fluid levels. Note diverticula of the sigmoid colon (thin arrows in **b**). Sagittal T2-weighted image (**c**) displays a fluid collection in the Douglas pouch (arrow). On axial DWI (*b* = 800 s/mm^2^) image (**d**), the fluid component of the adnexal masses demonstrates high signal intensity (arrowheads), consistent with restricted diffusion from purulent content

At MRI, TOA (Fig. 20) is usually T1-hypointense, but signal features are variable and often mixed depending on its haemorrhagic and protein content. The presence of a hyperintense rim along the inner wall of the collection has been recently described and attributed to the presence of granulation tissue with haemorrhage [41]. T2-weighted images demonstrate a heterogeneous, predominantly hyperintense mass with multiple thick low-signal septa. The increased signal intensity of the surrounding peritoneal fat on T2-weighted sequences with fat saturation corresponds to associated oedema. Septa, capsules of fluid collections, and surrounding inflammatory stranding demonstrate intense enhancement after intravenous gadolinium; these structures, along with the purulent content of the mass, display restricted diffusion on DWI sequences. Enhancement of the peritoneum and uterine ligaments is also present [37, 38, 41, 45].

The differential diagnosis from an adnexal tumour may prove very challenging. The cross-sectional appearance of a TOA may closely resemble those of ovarian epithelial malignancies, but it should be remembered that ovarian cancer is generally not associated with tubal dilation [41]. The exceedingly rare primary fallopian tube carcinoma (PFTC) manifests with characteristic symptoms (colicky pelvic pain, adnexal mass and serosanguineous vaginal discharge, collectively referred to as “Laztko’s triad”) only in 15% of patients [46]. Ma et al. have described three characteristics, helpful MR findings of PFTC: (a) a tubular- or sausage-shaped mass with a solid component showing variable, generally homogenous, T1-hypointensity, T2-hyperintensity and moderate enhancement after intravenous contrast agent administration; (b) hydrosalpinx; and (c) intrauterine fluid [47]. Hydrosalpinx is caused by both the partial obstruction of the tubes and the fluid produced by the neoplasm. Due to the patency of the fallopian tubes, the tubal content may be discharged into the endometrial cavity or the peritoneum with consequent temporary shrinkage of the mass and relief of pelvic pain. Serial imaging examination may demonstrate this morphological variability of the mass. Intrauterine fluid is quite specific of PFTC, occurring in up to 30% of cases [46]. On DWI, the solid component of the mass demonstrates higher signal intensity and lower ADC values compared to the normal ovarian tissue [48].

### Complicated and atypical PID forms

**Fig. 21:**
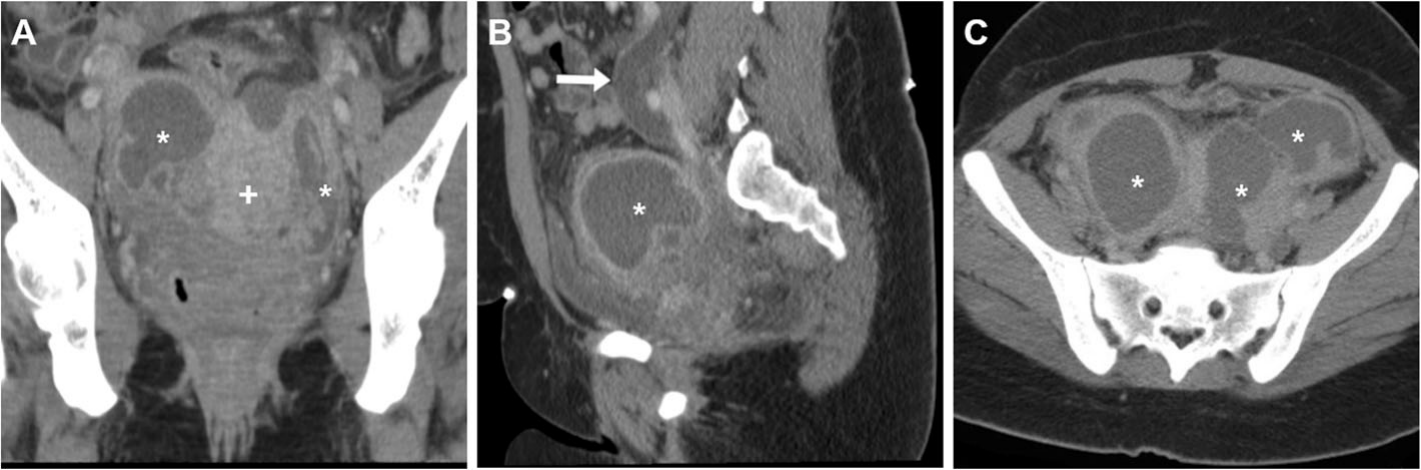
**a**–**c** Multiplanar CT images of an extensive PID in a 27-year-old African woman, complicated by multiple, confluent abscesses (asterisk) occupying the entire pelvis, that compress the uterus (plus sing) on the midline. Note hydronephrosis (arrow in **b**). Clinical and radiological suspicion of actinomycosis was not supported by microbiology samples

Occasionally, PID may spread even further in the pelvis forming peritoneal cavity abscesses (Fig. 21) or involve other adjacent organs such as the bowel, bladder and ureters. In these cases, recognition of anterior displacement of the broad ligament may be helpful to distinguish TOA from pelvic abscesses with a different origin. Furthermore, when faced with extensive pelvic infection, the diagnosis of actinomycosis should be suggested. Strongly associated with IUCD, *Actinomyces israelii* infection of the female genital tract characteristically spreads across soft-tissue planes, resulting in heterogeneous adnexal masses with small rim-enhancing hypoattenuating abscesses, complex masses in the peritoneal cul-de-sac and perirectal region, that commonly appear more solid, mass-like compared with usual TOA [49].

**Fig. 22:**
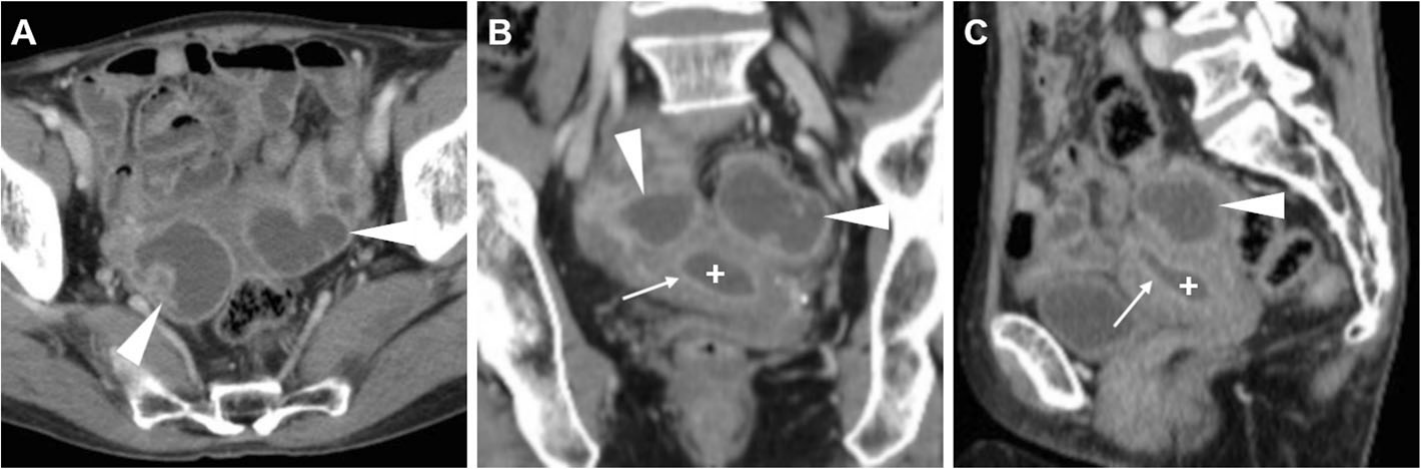
**a**–**c** Multiplanar CT images of genital involvement in a 58-year-old woman with respiratory tuberculosis, showing bilateral adnexal abscess-like enlargement (arrowheads) with peripheral enhancement, and dilated uterine cavity with thin endometrial enhancement (thin arrows) suggesting pyometra [adapted from Open Access ref. [50]]

Finally, in Western countries, genitourinary tuberculosis is increasingly encountered because of the spread of HIV infection and increasing immigration; the fallopian tubes are the commonest site of tuberculous involvement, which appears as bilateral pyosalpinx or TOA. Calcifications are uncommon, particularly in the acute phase. Coexistent endometritis appears as distended pus-filled uterine cavity and prominent endometrial enhancement (Fig. 22). Peritoneal and omental thickening, ascites and associated nodal or parenchymal involvement may help to suggest the diagnosis of tuberculosis, which may closely mimic peritoneal carcinomatosis [43].

### Vaginal infections

**Fig. 23:**
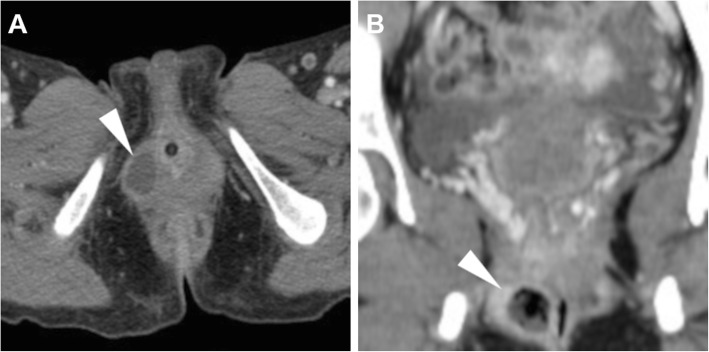
Two cases of vulvar abscesses (arrowheads) which required surgical incision. **a** Axial CT image of a small ovoid fluid collection with peripheral enhancement in the left-sided major labia. **b** Coronal CT image of an abscess with mixed fluid and gaseous content secondary to Bartholin gland infection

Vaginal abscesses appear as hypodense, thick-walled rim-enhancing lesions, which may arise from *Chlamydia*, *N. gonorrheae* or polymicrobial infection of the Bartholin gland (located at the posterolateral vagina) and may require surgical incision (Fig. 23). Alternatively, the infected Gartner cyst should be suggested when located in the anterolateral wall at the proximal third of the vagina, and differentiated from a urethral diverticulum [49].

## Conclusion

Although discouraged in childbearing-age women, CT often provides the first diagnosis of an unsuspected acute gynaecological disorder; therefore, familiarity with their imaging appearances is crucial to avoid missing or misinterpreting clinically important entities which may require surgical treatment. If clinical conditions and scanner availability permit, MRI is superior to CT for further characterisation of uterine abnormalities causing acute pain and genital bleeding. Additionally, cross-sectional imaging is helpful to elucidate the varied imaging appearances of PID and atypical genital infections.

## Data Availability

Data sharing is not applicable to this article as no new data were created or analysed in this study.

## Abbreviations

ADC: Apparent diffusion coefficient
CRP: C-reactive protein
CT: Computed tomography
DWI: Diffusion-weighted imaging
ED: Emergency department
EP: Endometrial polyp
FP: Follicular phase
HIV: Human immunodeficiency virus
IUCD: Intrauterine contraceptive device
LP: Luteal phase
MRI: Magnetic resonance imaging
PFTC: Primary fallopian tube carcinoma
PID: Pelvic inflammatory disease
TOA: Tubo-ovarian abscess
UI: Uterine inversion
US: Ultrasound
